# Sildenafil helps the beat go on

**DOI:** 10.1113/EP093206

**Published:** 2025-09-27

**Authors:** Jules C. Hancox

**Affiliations:** ^1^ Cardiovascular Research Laboratories, Bristol Medical School (THS) University of Bristol Bristol UK

**Keywords:** antiarrhythmic, arrhythmia, PDE5, premature ventricular complex, PVC, sildenafil

1

Antiarrhythmic drugs have long been used for arrhythmia termination and maintenance of normal cardiac rate and/or rhythm to reduce morbidity and mortality caused by cardiac arrhythmias. Limitations of existing antiarrhythmic compounds drive an ongoing search for improved drugs (Saljic et al., 2023). In broad terms the two main approaches to this are: (1) the development of new compounds and (2) the repurposing of existing drugs that were developed for use against other clinical conditions, but that are subsequently found to have antiarrhythmic effects (Saljic et al., 2023). A clear attraction of the repurposing route is that because the drugs in question have already been approved for human use, safety and toxicity data have already been obtained and early‐phase human trials can, in principle, be streamlined. R&D costs should also be lower for a repurposed drug than for an entirely novel chemical entity. Examples of drugs approved for other indications that may have antiarrhythmic potential include the respiratory stimulant and TASK‐1 channel inhibitor doxapram and the 5′‐AMP‐activated kinase activator and antidiabetic drug metformin: both drugs may have utility in the treatment of atrial fibrillation (Saljic et al., 2023).

While atrial fibrillation has received considerable attention from a drug development perspective, the identification of novel or repurposed small molecule drugs effective against ventricular arrhythmia(s) is of high importance, given the severe consequences that can follow disrupted ventricular rhythm. Enter sildenafil. Originally produced and marketed by Pfizer as Viagra and now additionally widely available as off‐patent generic formulations, sildenafil is an orally active phosphodiesterase‐5 (PDE5) inhibitor that is an effective vasodilator in erectile dysfunction and pulmonary hypertension, through inhibiting breakdown of cyclic guanosine monophosphate (Roy et al., 2023). Cardiomyocytes normally express a low level of PDE5 and this may be upregulated in disease states (Roy et al., 2023). In healthy volunteers, acute sildenafil blunts systolic responses to β‐adrenoceptor stimulation with dobutamine (Borlaug et al., 2005). In this issue of *Experimental Physiology* Hutchings and colleagues present an intriguing case report in which sildenafil use has been linked to suppression of premature ventricular complexes (PVCs; Hutchings et al., 2026), an action that would be anticipated to be antiarrhythmic.

The report arises from the treatment with sildenafil of a middle‐aged woman with Reynaud's phenomenon secondary to systemic sclerosis (Hutchings et al., 2026). Previous holter ECG monitoring had highlighted that she experienced ectopic beats, and echocardiography had demonstrated normal systolic function and a lack of structural abnormalities. However, she was found to exhibit two different types of PVC (denoted ‘early’ and ‘late’) that could be distinguished by their morphology and timing relative to the previous beat. Two periods of continuous cardiac monitoring were undertaken (Hutchings et al., 2026). Following a control measurement period, administration of sildenafil was associated with a near abolition of late PVCs and reduction of early PVC frequency. The drug was then discontinued and during this ‘washout’ period the occurrence of both early and late PVCs increased towards baseline. Sildenafil was then recommenced (increasing up to a dose of 50 mg, three times daily), and after 3 weeks a 4‐day period of cardiac monitoring was performed. No obvious changes to several key ECG parameters were seen with chronic sildenafil treatment, but the average PVC frequency was again decreased. Additionally, with sildenafil early PVCs occurred at higher heart rates than in control and their timing was altered: they occurred further from the T wave apex. Interestingly, the patient also exhibited some premature atrial complexes, and during sildenafil treatment these appeared to arise later in the cardiac cycle; however, their baseline frequency was low making firm conclusions regarding effects on their frequency difficult to make (Hutchings et al., 2026).

Definitively ascribing causality in individual case reports is challenging and for studies of *adverse* drug events, causality scales such the Naranjo method (Naranjo et al., 1981) or World Health Organization–Uppsala Monitoring Centre (WHO‐UMC) system can be employed. Several factors make it likely that sildenafil use was causally related to the potentially *beneficial* changes to PVCs in this study. First, the observed effects on PVC frequency were reversible on sildenafil washout. Second, the design of the study essentially built in ‘rechallenge’ and the second period of sildenafil administration was also associated with beneficial effects. Third, as the authors have highlighted, similar observations of reduced PVC frequency and delayed onset have been made for an in vivo sheep model of drug‐induced long QT syndrome (Hutchings et al., 2021). Taken together, these points strongly suggest that sildenafil treatment was responsible for the observed changes to the patient's PVCs.

It is noteworthy that PVCs from the patient studied here showed no ‘R‐on‐T’ phenomenon and no couplets or non‐sustained ventricular tachycardia was observed, as might occur in settings in which ventricular ectopics are linked to malignant arrhythmias (Ng, 2006). That said, the authors highlight that the two types of PVC likely arose from different sites in the ventricle; both types were beneficially affected by sildenafil. Further, in prior sheep work, both the R‐on‐T phenomenon and torsades de pointes were suppressed by sildenafil (Hutchings et al., 2021). Thus, while this case report does not directly study a documented arrhythmia, it is reasonable to conclude that the observed effects on PVCs strongly point towards antiarrhythmic potential of sildenafil. In sheep cardiomyocytes, sildenafil abolished delayed after‐depolarizations, decreased sarcoplasmic reticulum Ca^2+^ content and reduced SERCA activity and sarcolemmal L‐type calcium current (Hutchings et al., 2021). Such actions could plausibly underlie the effects of sildenafil on PVCs in this case report. Further in vitro work on cellular mechanisms of sildenafil antiarrhythmic action using wide‐ranging proarrhythmic challenges is warranted.

Sildenafil pretreatment has been shown to protect rat hearts against ventricular fibrillation linked to ischaemia–reperfusion, but at higher doses it increased the ventricular fibrillation incidence (Das et al., 2002). The dose of sildenafil for chronic treatment for the present case study was recommended by a rheumatologist not a cardiologist (Hutchings et al., 2026), and it would be interesting in the future both to determine (with a substantial patient cohort) the lowest daily dose that produces the kind of cardiac effects reported here and to extend the administration and measurement period to monitor long term effects. Further work could investigate effects on occurrence of human ventricular tachyarrhythmias.

Overall, this case report has significant sentinel value, providing a basis from which further human studies can be pursued to establish the settings and extent to which sildenafil may offer a novel, intracellular signalling based antiarrhythmic treatment approach.

## AUTHOR CONTRIBUTIONS

Sole author.

## CONFLICT OF INTEREST

The author declares he has no conflicts of interest.
